# The Internal Cranial Anatomy of a Female With Endocrine Disorders From a Mediaeval Population

**DOI:** 10.3389/fendo.2022.862047

**Published:** 2022-04-14

**Authors:** Anna Maria Kubicka, Philippe Charlier, Antoine Balzeau

**Affiliations:** ^1^Department of Zoology, Poznań University of Life Sciences, Poznań, Poland; ^2^PaleoFED Team, Unité Mixte de Recherche (UMR) 7194, Centre National de la Recherche Scientifique (CNRS), Département Homme et Environnement, Muséum National d’Histoire Naturelle, Musée de l’Homme, Paris, France; ^3^Laboratoire Anthropologie, Archéologie, Biologie (LAAB), Unité de Formation à la Recherche (UFR) des Sciences de la Santé, Université Paris-Saclay (UVSQ) & Musée du quai Branly - Jacques Chirac, Montigny-le-Bretonneux, France; ^4^Direction, Département de la Recherche et de L’Enseignement Musée du quai Branly - Jacques Chirac, Paris, France; ^5^Royal Museum for Central Africa, Department of African Zoology, Tervuren, Belgium

**Keywords:** gigantism, acromegaly, computed-tomography, endocast, skull thickness, frontal sinuses, sella turcica

## Abstract

Gigantism and acromegaly have been observed in past populations; however, analyses usually focus on the morphological features of the post-cranial skeleton. The aim of this study is to characterize the internal anatomical features of the skull (brain endocast anatomy and asymmetry, frontal pneumatization, cranial thickness, sella turcica size) of an adult individual from the 11-14th centuries with these two diseases, in comparison with non-pathological individuals from the same population. The material consisted of 33 adult skulls from a mediaeval population, one of them belonging to an adult female with endocrine disorders (OL-23/77). Based on the CT scans, the internal cranial anatomy was analysed. The sella turcica of OL-23/77 is much larger than in the comparative sample. The endocast of the individual OL-23/77 shows a left frontal/left occipital petalia, while the comparative population mostly had right frontal/left occipital petalias. The asymmetry in petalia location in OL-23/77 comes within the range of variation observed in the comparative population. The individual has high values for cranial thickness. The frontal sinuses of the specimen analysed are similar in size and shape to the comparative sample only for data scaled to the skull length. Enlarged sella turcica is typical for individuals with acromegaly/gigantism. The pattern of the left frontal/left occipital petalia in the specimen OL-23/77 is quite rare. The position of the endocranial petalias has not influenced the degree of asymmetry in the specimen. Despite the large bone thickness values, skull of OL-23/77 does not show any abnormal features. The skull/endocast relationship in this individual shows some peculiarities in relation to its large size, while other internal anatomical features are within the normal range of variation of the comparative sample.

## 1 Introduction

Gigantism and acromegaly are endocrine disorders whose differentiation has remained unclear long after its signs and symptoms were first described (1). At present, gigantism is related to excessive growth hormone secretion (GH) during childhood, while acromegaly results in excess GH in adulthood (2). Most individuals with gigantism and acromegaly exhibit physical and anthropological symptoms such as accelerated growth, elongated facial features, disproportionately large hands, arthritis, frequent headaches and excessive sweating (2, 3). According to epidemiological data, these two diseases are not common (4), and their frequency differs in present-day societies. In Spain and Belgium, the prevalence of acromegaly ranges from 34 to 40 cases per million (c.p.m.); in the UK, it is much higher at around 86 c.p.m., and in Northern Finland, in turn, it is only 0.34 c.p.m (5). Slight more frequent prevalence was reported in women (4); however, it seems that both sexes are equally affected. In turn, only about 200 reported cases of gigantism are known worldwide so far (6).

Several cases of gigantism and acromegaly have been reported in past human populations from the Third (2700 BC) (7), and Fifth Dynasties in Egypt (2494-2345 BC) (8), the Windmiller culture in California (2500-850 BC) (9), Mediaeval Greece (7^th^ century) (10), a post-Mediaeval cemetery in Turkey (11) a Jewish necropolis in Spain (7^th^-12^th^ centuries) (12), a late prehistoric village in Pottery Mound (14^th^-16^th^ centuries) (13), and ruins in New Mexico (14^th^-17^th^ centuries) (14). These studies on osteological material focus mostly on skeletal morphology, body height and coexisting pathological conditions such as joint diseases, ossification or periostitis (7–9, 15). Less often, research investigates the aspects of mortuary treatment and social perceptions (16). The medical literature describes more recent historical cases of patients with acromegalic and gigantism living in Europe, the USA and Russia in the 16^th^-19^th^ centuries (17, 18). These clinical descriptions usually focus on living patients and contain anthropometric data [e.g., body height and weight (19), information on physiological features (e.g., accelerated pulse (20), deep voice (19)], or cognitive abilities (19). In turn, autopsy reports of the historical cases describe anatomical features of the pituitary area or internal organs (21, 22). All these medical data made a valuable contribution to the first medical therapies of endocrine disorders (23).

Unfortunately, little attention is paid to craniometric analysis as well, as only a few studies have reported changes in the skull, and all have focused solely on living acromegalic patients. These cases show extensive frontal sinus pneumatization, enlarged sella turcica (24), large sphenoidal and maxillary sinuses, increased thickness of the frontal and occipital cranial vault (25), and reduced posterior fossa (26). Acromegaly starts to develop after epiphyses fusion; therefore, the cranial cavity is usually not enlarged due to the already ossified centres, with simultaneous changes in the facial bones and mandibular lengthening (27). The human skull is an integrated structure where the relationship between the facial skeleton (i.e. the zygomatic processes, nasal, lacrimal and maxillary bones), basicranium and cranial vault show a uniform pattern of integration (28). Therefore, the analysis of the internal cranial anatomy (i.e. endocast morphology, frontal pneumatization) in individuals with endocrine disorders is interesting in the following aspects.

First of all, the endocast size of an individual with gigantism and acromegaly may provide new information in the context of the skull morphological integration, e.g. indicate whether changes in the facial bones may affect the cranial vault despite their ossified centres. Another important aspect is whether the larger endocranial volume and elongated facial features of a person with endocrine disorders result in a different pattern of cranial thickness to that in non-pathological individuals. Previous research shows increased thickness of the cranial vault, but investigated only the frontal and occipital bones (25). Other features of the internal cranial morphology, such as asymmetry is also worth investigation in order to check whether endocrine disorders are associated with an atypical pattern of endocast asymmetry. Lateralization of brain functions in humans is related to their high cognitive abilities as each hemisphere has a different specialization (29, 30). Therefore, considering that patients with acromegaly can exhibit mental disorders such as dementia, affective and mild cognitive disorders (31, 32), and gigantism can be related to poor mental development (33), we can suspect different endocast asymmetry than in healthy individuals. The last aspect concerns the frontal sinuses, which are characterized by considerable morphological variability (34, 35). There is a strong association between the brain asymmetry and the shape and extension of the frontal sinuses (35). Therefore, the analysis of the morphometric data on the frontal pneumatization may improve the understanding of the effect of excessive secretion of GH on the skull growth factor.

Excessive GH secretion may occur as a wide spectrum of heterogenous disorders therefore, each case of this endocrinological condition, even historical, provides new information on the pathological features. Nevertheless, very few cases of gigantism and acromegaly that is a more common endocrine disorder have been described in the palaeopathological literature. That is why this study presents a rare case of an adult individual with both gigantism and acromegaly from a mediaeval population in Poland. For this purpose, we decided to focus on craniometric data that is rarely studied. The size and asymmetry of the endocast, frontal pneumatization, cranial thickness and sella turcica size of an adult female with endocrine disorders were compared with a large sample of adults without visible pathological conditions from the same mediaeval group. CT images were used with modern anthropological methods such as three-dimensional reconstructions of endocasts and frontal sinuses and a tomographic map of variation in the total bone thickness. Since the pattern of cranial features is established early in ontogeny, this case study may show whether progressive endocrine disorders result in atypical features of the internal cranial anatomy. Moreover, documenting an individual from a pre-modern industrial society with acromegaly and gigantism using medical facilities contributes to our knowledge of normal human variation.

## 2 Material and Methods

The material consists of an adult individual with gigantism and acromegaly (OL-23/77) and 32 adults (16 females and 16 males) from a mediaeval population dated to a period from the end of 11^th^ century to the 13-14^th^ centuries (36, 37). The settlement is located in Ostrów Lednicki (Poland), near the eastern shore of Lake Lednica. According to archaeological and anthropological records, the subsistence economy of this population was based on agriculture, but other activities such as stockbreeding or hunting small mammals and birds were also present (38, 39).

The sex and age of 33 adult individuals from Ostrów Lednicki were assessed based on morphological changes in the skull and pelvis. The sex was assessed using the morphology of the supraorbital ridges, glabella region, orbits, mastoid processes, occipital condyles, greater sciatic notch, subpubic concavity, and ischiopubic ramus. Age at death was assessed based on the modification of the pubic symphysis and cranial suture closure (40–43). The skeleton of an individual OL-23/77 was 208 cm long *in situ* and showed characteristic morphological features of an individual with endocrine disorders. Further examination showed that this was a female aged 25 to 30 at the time of death with a body stature estimated at 215.5 cm (44). Based on osteological, radiological and CT examinations, the skeleton was diagnosed as excessively long and massive but with normal bone proportions that indicate gigantism in the early years, and with an elongated and prognathic mandible, enlarged vertebrae and thick bones that indicate acromegaly in adulthood (16, 33). Furthermore, based on the X-rays and CT-scans, the OL-23/77 skeleton shows pituitary lesion, degenerative joint disease, Schmorls’s nodes [i.e. pathological condition of vertical herniation of intervertebral discs into the neighbouring vertebrae (45)] in almost all vertebrae and healed fractures of the humerus and tibia. Other pathological conditions indicating a developmental disorder of the external auditory canal and sclerotization of the left temporal bone were also found (46). The skeleton is preserved in good condition, with a few missing bones such as the 4^th^ cervical vertebra, two metacarpal and 5 metatarsal bones (33). The comparative sample contains 32 adult individuals from Ostrów Lednicki that did not show any pathological changes (e.g. osteophytes, porosity, trauma) and were characterized by a good state of preservation of the skull. The average body stature of the comparative sample was 162.10 cm ( ± 7.37).

### 2.1 Methods

#### 2.1.1 3D Reconstruction

The skulls of 33 adult individuals from Ostrów Lednicki were scanned using 32-slice computed tomography (Siemens SOMATOM Sensation) in the cranio-caudal position with the standard protocol [0.625 slice thickness, 60 Vox energy, Balzeau et al. (47)]. Next, for each individual, a three-dimensional (3D) reconstruction of the skull was prepared using InVesalius software (ver. 3.1.1). Based on the reconstructed skulls, 3D endocast models were then created using the *endomaker* algorithm in R software (48).

#### 2.1.2 Measurements of the Sella Turcica

Based on the 3D skull reconstruction, each sella turcica was measured using GomInspect software (ver. 2.0.1). According to Hawkins (49), three dimensions of this bone structure were measured: length (antero-posterior diameter), width (medio-lateral diameter) and height (supero-inferior).

#### 2.1.3 Endocast

**Figure 1:**
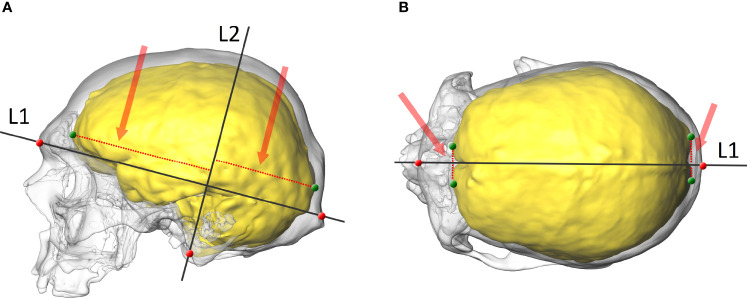
The skull and endocast of an adult female with endocrine disorders (OL-23/77). **(A)** lateral view with two projected lines (L1 and L2), points on the skull (red) and the endocast (green), and the vertical dimensions of the frontal and occipital lobes (red dotted line and arrow). **(B)** superior view with a projected line (L1), points on the skull (red) and the endocast (green), and the lateral dimensions of the frontal and occipital lobes (red dotted line and arrow).

The anatomy of the OL-23/77 endocast was analysed from the 3D reconstruction. In order to explore the relative position of the endocranial petalias, a tested and validated protocol was used (50). Initially, three anatomical points on each skull (i.e. glabella, inion and basion) were digitized. These in turn, were used to construct two lines, the first (L1) connecting the glabella and inion, the second (L2) starting at the basion and passing perpendicularly through L1 (**Figure 1**). Then, four points were digitized in the following locations: two points on the most anterior part of the left and right frontal lobes, and two points on the most posterior part of the left and right occipital lobes (**Figure 1**). Six dimensions between the points located on the lobes and two lines (L1 and L2) were taken to quantify the location of the most protruding points on the frontal and occipital lobes in the antero-posterior, vertical and lateral views (**Figure 1**). The maximum length of the skull was then measured.

The cranial vault thickness of OL-23/77 was computed across the whole vault using the Surface Thickness module in Avizo 7 software (FEI, Hillsboro, Oregon). A 3D tomographic map of variation in the total bone thickness was computed based on the 3D model of the exo- and endocranial skull surfaces. Next, the map was completed using a 2 to 22 mm colour scale from white to yellow, where white indicates bone thickness close to 2 mm and yellow indicates bone thickness close to 22 mm.

#### 2.1.4 Frontal Sinuses

The 3D models of the frontal sinuses were manually reconstructed using multiple threshold values as a function of the modification of the grey values of the tissues in the area of frontal pneumatization. The segmentation was performed using Avizo 7 software (FEI, Hillsboro, Oregon). Next, the 3D models of the skulls with the reconstructed frontal sinuses were positioned in the Frankfurt plane (i.e. a plane formed with a horizontal line running from the superior edge of the external auditory canal to the inferior border of the orbit) to measure dimensions in the anterior, superior and vertical orientations (**Table 1**). As the frontal sinuses can be insufficiently preserved in the inferior extension, the variables were selected to ensure the measuring of parts that are usually well preserved. These measurements define the maximal extension of the frontal sinuses in 3D, including bilateral data for the two sinuses. The bilateral measurements (i.e. AL, AR, SL and SR) and two antero-posterior dimensions in the left lateral view (AP, AP2) were combined into three final parameters: 2A, 2S and 2AP (**Table 1**). A relative value was then calculated for each final parameter of the frontal sinuses by scaling measurements to the cube root of the volume.

**Table 1:** Description of the measurements of the frontal sinuses in 3D.

Parameter	View	Description	Final parameter
W	anterior	the maximal lateral extension of the pneumatisation	W
H	anterior	the maximal height of the frontal sinus	H
AL	anterior	the maximal length of the left frontal sinus	2A (combined AL and AR
AR	anterior	the maximal length of the right frontal sinus	
SL	superior	the maximal medio-lateral extension of the left frontal sinus	2S (combined SL and SR)
SR	superior	the maximal medio-lateral extension of the right frontal sinus	
AP	left lateral	the length from the most anteriorly protruding point of the left sinus to the most posterior point in an horizontal direction	2AP (combined AP and AP2)
AP2	left lateral	the length from the most anterior point of the sinus to the maximal supero-posterior extension of the sinus	
RCV		cube-root of the volume of both frontal sinuses	RCV

### 2.2 Statistical Analysis

#### 2.2.1 Sella Turcica

For each individual, the volume [mm^3^] of the sella turcica was calculated using the formula: (width × length × height)/2 (49). Next, descriptive statistics were calculated separately for the comparative sample and OL-23/77.

#### 2.2.2 Asymmetry of the Endocast

In order to quantify asymmetry in petalia location, different indices were calculated in the study. Signed asymmetry (DA) is present when the difference between the left and right sides of the endocast is greater than zero. This is calculated using the following equation: (R – L), where R is a dimension of the right petalia, and L is a dimension of the left petalia. Positive values of this index indicate asymmetry towards the right side, while negative values mean asymmetry towards the left side of the endocast. The second index was in absolute asymmetry (AA), which indicates the degree of directional asymmetry but without the directional bias and is calculated using the equation (maximum value – minimum value). The indicator of directional asymmetry (AQ) was also calculated using the formula (R-L)/((R+L)/2) (51), Indicators of DA and AA were calculated for all six dimensions to explore the relative position of the endocranial petalias, while AQ was calculated only for the two dimensions quantifying frontal and occipital petalias in the lateral position.

#### 2.2.3 Regression Between Endocast and Skull Length

The regression between the length of the skull and the position of the frontal and occipital poles was calculated using bivariate linear regression (RMA model) to summarize the relationship between variables. This model was calculated only with the comparative sample and is better than alternative regression models when outliers are expected (52).

#### 2.2.4 Pneumatization of the Frontal Bone

In order to analyse the pneumatization of the frontal bone, descriptive statistics were calculated separately for OL-23/77 and the rest of the sample.

## 3 Results

**Table 2**. shows the descriptive statistics of the sella turcica parameters. OL-23/77 is characterized by larger measurements (i.e. length, width, height and volume) of the sella turcica than the comparative sample.

**Table 2:** Descriptive statistics of the sella turcica measurements.

	Length [mm]	Width [mm]	Height [mm]	Volume [mm^3^]
*OL-23/77 (n=1)*				
Giant	14.10	21.22	8.72	1304.52
*Individuals from Ostrów Lednicki (n=32)*				
Min	7.12	10.07	2.52	163.44
Mean-SD	8.81	11.70	3.57	218.66
Mean	10.17	13.61	5.27	361.56
Mean+SD	11.53	15.52	6.97	504.46
Max	12.90	17.75	8.61	650.62
SD	1.36	1.91	1.70	142.90

**Figure 2:**
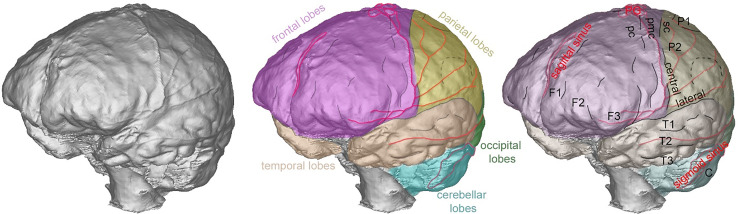
Left anterior views of the endocast of OL-23/77 showing the extension and position of the frontal lobes (pink), parietal lobes (yellow) temporal lobes (orange), occipital lobes (green), cerebellar lobes (blue), some aspects of the venous system in red (middle meningeal system, main venous sinuses and pacchionian granulations – noted PG), the position of the central and lateral sulci as well as several smaller sulci (in black) delimiting the three frontal convolutions (noted F1 to F3), the first and second parietal convolutions, the three temporal convolutions and anatomo-functional areas: premotor cortex (pc), primary motor cortex (pmc), somatosensory cortex (sc).

The endocast of OL-23/77 does not exhibit any abnormal features. There is no alteration of its global shape, no sign of pathologies on the endocranial surface or in the extension of the sinus drainage pattern of the middle meningeal system. The analysis of the endocast anatomy shows that the superior sagittal sinus is visible nearly all along with its extension between the frontal and parietal lobes, continuing posteriorly into the right lateral sinus (**Figure 2**). The right lateral and sigmoid sinuses are larger than those visible on the left side. The middle meningeal system is well developed on both sides with a very well developed coronal segment and a well-marked posterior inferior branch (**Figure 2**). The development is quite similar on both sides, including the presence of several anastomoses on the parietal lobes. We did not detect any potential alteration in the structure of the brain as identified by the position of the sulci (**Figure 2**). Important anatomical and functional areas such as the premotor cortex, the primary motor cortex and the somatosensory cortex exhibit normal morphology, disposition and extension. Similarly, the third frontal convolutions and the supramarginal gyrus are of normal size and shape. The glabella-inion length of OL-23/77 is 208.2 mm.

Concerning the petalias, the endocast of OL-23/77 shows a left frontal/left occipital petalia. In the sample from Ostrów Lednicki comprising 32 individuals, the right frontal/left occipital is the most frequent, visible in 27 specimens. No left frontal/left occipital is visible in any specimen, but one specimen has a left frontal petalia. In terms of metric data (**Table 3**), the OL-23/77 skull values are within the range of variation observed for each parameter in the comparative sample. Values for the vertical petalia are higher than those for the antero-posterior petalia in the comparative sample, and values for the lateral petalia are even higher.

**Table 3:** Directional and absolute asymmetry of the frontal and occipital petalias.

	Frontal petalia				Occipital petalia			
	Antero-posterior	Vertical	Lateral		Antero-posterior	Vertical	Lateral	
	DA (AA)	DA (AA)	DA (AA)	AQ	DA (AA)	DA (AA)	DA (AA)	AQ
*OL-23/77 (n=1)*								
Giant	-1.08 (1.08)	-2.53 (2.53)	7.10 (7.10)	0.57	-0.36 (0.36)	-5.55 (5.55)	13.85 (13.85)	0.25
*Individuals from Ostrów Lednicki (n=32)*								
Min	-1.63 (0.01)	-7.86 (0.53)	-9.78 (0.03)	-1.07	-4.58 (0.06)	-8.42 (0.51)	-15.20 (0.08)	-0.43
Mean-SD	-0.53 (0.23)	-4.64 (1.23)	-4.64 (0.84)	-0.49	-3.05 (0.52)	-5.01 (0.33)	-5.02 (2.38)	-0.12
Mean	0.43 (0.80)	-0.10 (3.73)	-0.09 (3.58)	-0.04	-1.17 (1.79)	-0.51 (3.34)	3.35 (7.40)	0.06
Mean+SD	1.39 (1.47)	4.44 (6.23)	4.46 (6.31)	0.40	0.71 (3.06)	4.00 (6.34)	11.72 (12.42)	0.24
Max	3.02 (3.02)	9.85 (9.85)	8.29 (9.78)	0.81	4.47 (4.58)	13.88 (13.88)	22.55 (22.55)	0.48
SD	0.96 (0.67)	4.54 (2.50)	4.55 (2.74)	0.45	1.88 (1.27)	4.50 (3.01)	8.37 (5.02)	0.18

DA, signed asymmetry; AA, absolute (unsigned) asymmetry; AQ, indicator of directional asymmetry of the lateral petalias; Giant, an individual with gigantism and acromegaly from Ostrów Lednicki; n, number of individuals; Min, minimal observed value; Max, maximal observed value; Mean - SD, mean value minus standard deviation, Mean - mean value, Mean + SD - mean value plus standard deviation; SD, standard deviation.

**Figure 3:**
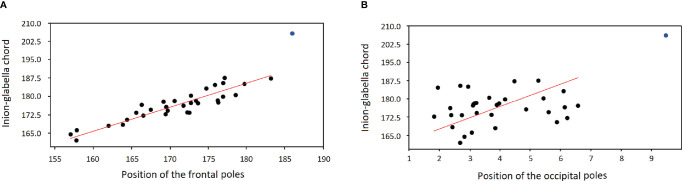
Bivariate linear regression (RMA model) between the length of the skull (Y axis; Glabella-Inion length) and the position of the frontal **(A)** and occipital poles **(B)** on this axis (X axis, i.e. the position of the mean projection of the left and right poles on the Inion-Glabella chord). The blue dot shows the position of OL-23/77.

**Figure 3A** shows the regression (RMA) between the length of the skull (inion-glabella chord) and the position of the frontal poles. The OL-23/77 skull has higher values for both parameters and does not follow the regression observed in the comparative sample (32 adult individuals from Ostrów Lednicki). For the position of the occipital poles, the skull of the OL-23/77 is also large but only slightly continuing the regression line calculated for the comparative sample (**Figure 3B**). This means that the frontal poles are in a more posterior position than expected for an individual of this size. In contrast, the occipital poles are in a slightly more posterior position than expected.

**Figure 4:**
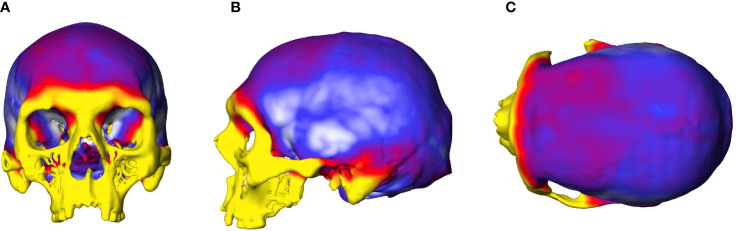
Graphic representation of the cranial thickness of the female with gigantism and acromegaly. **(A)** anterior view of the cranium, **(B)** lateral view of the cranium, **(C)** superior view of the cranium. Colours indicate the variation in bone thickness, from white (2mm) to dark red/yellow (22 mm).

The OL-23/77 skull has quite high values for bone thickness. The frontal bones are thicker overall than the parietal bones, but with thinner areas in the region of the temporal squama, at the third frontal convolution (**Figure 4**). Note the strong and thick reliefs in the anterior part of the frontal bone, related to the high bone thickness values as illustrated by the presence of a yellow area. This also corresponds to the area where the frontal poles are located on the endocranial surface. The occipital poles are located around the middle of the lambda-inion chord, where bone thickness is less pronounced than in the region of the inion. We also observe that the endinion is located below the inion, but this is a frequent pattern in *Homo sapiens* (53).

Concerning pneumatization of the frontal bone, the OL-23/77 specimen exceeds the range of variation observed in the comparative sample for nearly all the variables analysed when absolute values are considered (**Table 4**). In the case of relative measurements (scaled to the cube root of the volume of pneumatization), the values for the specimen are comparable to the rest of the mediaeval population. This is informative because we observed that bone thickness in this area is higher than expected for an individual of this size, indicating that the frontal sinuses have not developed as much as the available space in the bone would have allowed. We also observed that the maxillary sinuses and sphenoid pneumatization in this specimen are of normal size and shape.

**Table 4:** Morphometric data for the pneumatization of the frontal bone.

	W [mm]	H [mm]	2A [mm]	2S [mm]	2AP [mm]	RCV [mm]	Wr [mm]	Hr [mm]	2Ar [mm]	2Sr [mm]	2Apr [mm]
*OL-23/77 (n=1)*											
Giant	80.3	41.4	88.9	90.2	75.8	30.2	265.8	137.0	294.3	298.4	250.9
*Individuals from Ostrów Lednicki (n=32)*											
Min	0	0	0	0	0	0	0	0	0	0	0
Mean-SD	24.8	13.0	23.8	23.4	12.8	9.2	195.2	113.3	197.1	196.5	118.7
Mean	41.4	22.2	41.1	42.3	25.7	14.6	269.6	146.3	265.3	269.9	164.7
Mean+SD	58.0	31.4	58.4	61.2	38.6	20.0	344.0	179.3	333.5	343.3	210.7
Max	72.3	50.4	79.4	87.7	70.6	26.2	453.3	200.5	366.2	410.3	269.5
SD	16.6	9.2	17.3	18.9	12.9	5.4	74.4	33.0	68.2	73.4	46.0
V*	40.1	41.6	42.2	44.8	50.6	36.7	27.7	22.6	25.8	27.3	28.1

W, maximal lateral extension of the frontal sinuses; H, maximal height of the frontal sinuses; 2A, the combined maximal length of the left and right frontal sinuses; 2S, the combined maximal length of the left and right frontal sinuses; 2AP, the combined length of the left and right frontal sinuses from the most anterior point to the most posterior point; RCV, cube-root of the volume of the frontal sinuses; Wr, relative maximal lateral extension of the frontal sinuses scaled to the cube root of the volume; Hr, relative maximal height of the frontal sinuses scaled to the cube root of the volume; 2Ar, the combined relative maximal length of the left and right frontal sinuses in anterior view scaled to the cube root of the volume; 2Sr, the combined relative maximal length of the left and right frontal sinuses in superior view scaled to the cube root of the volume; 2Apr, the combined relative length of the left and right frontal sinuses from the most anterior point to the most posterior point scaled to the cube root of the volume; n, number of individuals; Min, minimal observed value; Max, maximal observed value; Mean - SD, mean value minus standard deviation; Mean, mean value; Mean + SD, mean value plus standard deviation; SD, standard deviation; V*, coefficient of variation corrected for small sample size.

## 4 Discussion

The morphological data of an adult individual with endocrine disorders (OL-23/77) were analysed in relation to non-pathological individuals from the same mediaeval group in Poland. The cranial length of OL-23/77 is similar to skull measurements reported in earlier archaeological studies and current patients with acromegaly (7–9, 11, 26). We were unable to analyse the cortical thickness of the cortex or variation in grey matter volume that is relevant for the cognitive abilities (54) as we investigated bone remains. Therefore we focus on the internal anatomy and its features, such as the size and asymmetry of the external endocast structures of the OL-23/77 sample, which are used in palaeoanthropology for the reconstruction of cognitive abilities (47, 55). The endocast anatomy of an adult female with endocrine disorders (i.e. acromegaly and gigantism) exhibits no signs of pathology. Moreover, the middle meningeal system and anatomo-functional areas (premotor, primary motor and somatosensory cortexes) of this individual (OL-23/77) show normal development and morphology, as in other non-pathological individuals from the same population that were used for comparison.

It was possible to measure and calculate the volume of the sella turcica in all collected individuals from the analysed mediaeval population. The sella turcica of OL-23/77 was enlarged (1304.52 mm^3^) compared to the comparative sample (mean: 361.56 mm^3^) and with an asymmetrically eroded floor of the bone. The large size and lytic destruction of the sella turcica in the analysed individual (i.e. OL-23/77) may result from the pressure erosion of an intrasellar tumour (49, 56). The calculated sella turcica volume of OL-23/77 falls within the range of other cases of gigantism and acromegaly (11, 49). The anatomical features of the skeleton and the large volume of the sella turcica confirm the earlier anthropological assessment, the presence of endocrine disorder in OL-23/77. The timing of GH excess determines the type of endocrine disorder, i.e. the occurrence of enlargement before and after the epiphyseal closure leads to gigantism or acromegaly, respectively. In turn, the onset secretion of GH in childhood and continuation into adulthood causes the coexistence of these two endocrine disorders (49, 56). The age at the time of death of the analysed female ranged from 25 and 30 years therefore gigantism is probable, as the body stature and elongated long bones suggest the excessive secretion of growth hormones early in life. Thick bones and enlarged and prognathic mandible of OL-23/77 might also indicate acromegaly. However, without genetic studies on gene mutations (e.g., AIP, PRKAR1A, GPR101, GNAS, MEN1, CDKN1B, SDHx, MAX (57), it is difficult to assess whether OL-23/77 was affected by gigantism and acromegaly or just one of these diseases. Especially, since some anatomical features such as elongated bones and high body stature, typical of gigantism, may also occur in individuals with acromegaly (56). Therefore, we suggest treating this case as an individual with gigantism/acromegaly.

Since the relationship between features of the brain torque (frontal/occipital petalia, bending and shift), cognitive function and mental health is significant (58), the pattern of endocast asymmetry in this study was investigated. The OL-23/77 sample shows a left frontal/left occipital petalia, a pattern that is quite rare in *Homo sapiens* but occurs more often in females (58–60). In turn, 27 out of 32 specimens from the comparative sample exhibit a right frontal/left occipital petalia, which is the most common pattern in *Homo sapiens* (61–63). Based on the lateral and vertical frontal and occipital petalias, the orientation of the asymmetries observed in OL-23/77 does not follow the classic pattern, but it is not unexpected and is certainly not related to the large size of the specimen. Both asymmetry indices (DA and AA) are high in the sample (OL-23/77); however, the values are within the range (i.e. minimum and maximum values) of the comparative samples. This confirms the previous findings that asymmetry is not the result of allometric scaling (59, 64).

The high cranial thickness values of OL-23/77 confirm the previous results indicating significantly thicker cranial vaults in acromegalic patients than in non-pathological individuals (65, 66). This could therefore be a consequence of the effects of GH on bone growth and the large size of the skull. However, the frontal bones are much thicker than the parietal bones of OL-23/77, possibly due to the timing of an endocrine disorder and the faster growth of the neurocranial bones than of the facial skeleton (67). The topographic mapping of vault thickness we obtained shows, however, that the pattern observed in the sample (OL-23/77), where the frontal bones are thick and the temporal squama is thin, is the same as in non-pathological individuals (68, 69). Moreover, OL-23/77 shows an unusually high position of the orbits in the skull that is typical for patients with gigantism (70). Perhaps, the position and high thickness of the frontal bones limit the position of the frontal lobes. On the other hand, thick parietal bones may have a slight impact on the parietal lobes of OL-23/77 therefore the correlation between the skull length and the position of the occipital poles of the sample (OL-23/77) follows the regression line calculated for the comparative sample.

Medical studies focus on pneumatization of the skull but usually in the context of incomplete sphenoid sinuses, which can complicate transsphenoidal surgery performed on patients with acromegaly and gigantism (3, 70). In historical cases of these two endocrine disorders, the pneumatization of other bones of the skull is also analysed, but in the light of their presence and size. Pneumatization of the frontal sinuses was found to be present in two individuals with signs of gigantism and acromegaly from a Late Holocene site in Central California (9) and in historic New Mexico (13). Both of these studies emphasize the enlargement of the frontal sinuses, which is consistent with the measurements obtained in our study where the absolute values for the frontal sinuses of OL-23/77 exceed the range of variation observed in the comparative sample from Ostrów Lednicki. However, when the scaled frontal pneumatization measurements were taken into account, OL-23/77 falls within the range of variation observed in the comparative group. This may mean that the frontal sinuses follow the developmental trajectory of the skull so that their size is the result of allometric scaling. We observed that the frontal sinuses have not developed in the large areas available, which relates to the unexpected thickness of the frontal bone in these areas. This might be related to arrested propagation of pneumatization before the frontal superstructures had attained their full development. This kind of observation is of interest in view of the as yet poorly understood causes of development of the sinuses.

Our study shows that the skull of OL-23/77 has the classic features observed in *H. sapiens* during previous studies in terms of endocast asymmetry, cranial vault thickness and frontal sinuses (35, 71, 72). Therefore, the internal anatomy of the adult female (OL-23/77) is similar to individuals without endocrine disorders. We did not observe any signs of pathology or abnormality of the brain, as the main anatomical and functional areas visible on the endocast exhibit a normal size, shape and expression. There is a relationship between brain torque and cognitive abilities (58); based on our results, it is not possible to confirm the earlier assumption of cognitive impairment of the female examined (33). Although the previous research suggested the presence of cognitive dysfunctions in acromegaly patients (73, 74) possibly due to the positive correlation of GH and the severity of cognitive impairments (75), the latest results of the neuropsychological tests and brain cortical thickness do not indicate significant differences between acromegaly patients and the general population (54). That is why the assessment of the cognitive abilities of individuals with endocrine disorders (i.e. gigantism and acromegaly) should be carried out with caution, especially when examining human remains. Nevertheless, this case study broadens our knowledge about the occurrence of gigantism and acromegaly in past populations, when the causes of these endocrine disorders were unknown, as well as provides information about variation in poorly investigated cranial traits.

## Data Availability Statement

The data supporting the conclusions of this article are available on request from the authors.

## Ethics Statement

Ethical review and approval was not required for the study on human participants in accordance with the local legislation and institutional requirements. Written informed consent for participation was not required for this study in accordance with the national legislation and the institutional requirements.

## Author Contributions

AMK, contributions to design, acquisition of data, data analysis and interpretation, writing of the manuscript, and approval of the article. PC, paleopathological analysis and interpretation, approval of the article. AB, contributions to design, data analysis and interpretation, writing of the manuscript, and approval of the article. All authors contributed to the article and approved the submitted version.

## Funding

Research of the first author (AMK) is financed through the Polish Ministry of Science and Higher Education (506.511.09.00) and the Polish National Agency for Academic Exchange (PPB/BEK/2018/1/00390/U/00001/02). Research of the last author (AB) on brain anatomy is financed through the PaleoBRAIN project by the ANR (Agence nationale de la recherche, grant ANR-20-CE27-0009).

## Conflict of Interest

The authors declare that the research was conducted in the absence of any commercial or financial relationships that could be construed as a potential conflict of interest.

## Publisher’s Note

All claims expressed in this article are solely those of the authors and do not necessarily represent those of their affiliated organizations, or those of the publisher, the editors and the reviewers. Any product that may be evaluated in this article, or claim that may be made by its manufacturer, is not guaranteed or endorsed by the publisher.
